# Usability and utility of eHealth for physical activity counselling in primary health care: a scoping review

**DOI:** 10.1186/s12875-020-01304-9

**Published:** 2020-11-06

**Authors:** Apichai Wattanapisit, Titiporn Tuangratananon, Sanhapan Wattanapisit

**Affiliations:** 1 School of Medicine, Walailak University, Thasala, Nakhon Si Thammarat, Thailand; 2 Walailak University Hospital, Thasala, Nakhon Si Thammarat, Thailand; 3 International Health Policy Program, Ministry of Public Health, Muang, Nonthaburi, Thailand; 4 Thasala Hospital, Thasala, Nakhon Si Thammarat, Thailand

**Keywords:** counselling, eHealth, physical activity, primary health care

## Abstract

**Background:**

Physical activity (PA) counselling is an effective approach to promote PA in primary health care (PHC). Barriers to PA counselling in PHC include time constraints, lack of knowledge and skills of providers, and systemic barriers. Using electronic health (eHealth) has the potential to promote PA. This scoping review aimed to identify usability and utility of eHealth for tailored PA counselling introduced in PHC settings.

**Methods:**

A scoping review included primary research articles. The authors systematically searched six databases (Cochrane Library, CINAHL Complete, Embase, PubMed, Scopus and Web of Science) from the inception of the databases. The search terms consisted of three search components: intervention (PA counselling), platform (eHealth), and setting (PHC). Additional articles were included through reference lists. The inclusion criteria were research or original articles with any study designs in adult participants.

**Results:**

Of 2501 articles after duplicate removal, 2471 articles were excluded based on the title and abstract screening and full text review. A total of 30 articles were included for synthesis. The eHealth tools had a wide range of counselling domains as a stand-alone PA domain and multiple health behaviours. The included articles presented mixed findings of usability and utility of eHealth for PA counselling among patients and providers in PHC settings. Technical problems and the complexity of the programmes were highlighted as barriers to usability. The majority of articles reported effective utility, however, several articles stated unfavourable outcomes.

**Conclusions:**

eHealth has the potential to support PA counselling in PHC. Facilitators and barriers to eHealth usability should be considered and adapted to particular settings and contexts. The utility of eHealth for promoting PA among patients should be based on the pragmatic basis to optimise resources.

**Supplementary Information:**

The online version contains supplementary material available at 10.1186/s12875-020-01304-9.

## Background

Physical activity (PA) is associated with several health benefits, including a reduction in risks of several medical conditions and premature mortality [1–3]. PA is one of the World Health Organization (WHO)'s important aspects in public health [4]. WHO has launched the Global Action plan on Physical Activity (GAPPA) 2018–2030 to create: (i) active society; (ii) active environments; (iii) active people; and (iv) active systems [5]. Implementing and strengthening systems to increase PA and reduce sedentary behaviour (SB) in healthcare sectors is one of the 20 policy actions suggested in the GAPPA [5]. The suggested action in healthcare settings to promote PA is the counselling process embedded within healthcare facilities. According to the proposed action, characteristics and roles of primary health care (PHC) systems (e.g. comprehensiveness, coordination first contact, cost-effectiveness) [6] are supposed to be a suitable setting for PA promotion [7, 8].

PA counselling is an approach to promote PA in PHC settings. PA counselling contains several processes: the assessment of current PA levels, advice on increasing PA; agreement to an individualised plan for PA; assistance in pertinent strategies to achieve PA goals; and arrangement for follow-ups [8, 9]. A systematic review and meta-analysis revealed that PA counselling by primary care providers is an effective tool to modify patients’ behaviours [10]. However, characteristics of PHC are diverse across countries, in terms of policies, resources, and strengths [11–13]. Implementing PA counselling in PHC is challenging. Barriers to PA counselling in PHC include time constraints, lack of knowledge and skills, and systemic barriers [14–17].

A study demonstrated that an electronic-based PA counselling system may be feasible for promoting PA among patients with chronic diseases [18]. Electronic health (eHealth), mobile health (mHealth), or electronic-based interventions have the potential to promote PA in PHC [19–21]. Although previous systematic reviews presented the effects of eHealth interventions on PA, they did not focus on PHC settings [22, 23]. To the best of our knowledge, using eHealth or electronic-based PA counselling systems in PHC and their outcomes vary in different PHC settings. The aim of this scoping review is to identify usability and utility of eHealth for tailored PA counselling introduced in PHC settings.

## Methods

The authors conducted this scoping review following the PRISMA extension for scoping reviews (PRISMA-ScR) [24].

### Search methods

The authors performed a systematic search in six databases: Cochrane Library, CINAHL Complete, Embase, PubMed, Scopus and Web of Science. The search included published articles from the inception of the databases to 16th January 2020. The search terms consisted of three search components: intervention (PA counselling), platform (eHealth), and setting (PHC). The search strategy is presented in Table 1. The filter function of each database was used to recruit articles published in English. All articles found from the databases were transferred to Endnote X4 citation manager (Thomson Reuters, Toronto, ON, Canada).

**Table 1:** Search terms

Search component	Search term
Intervention	(“physical activity” OR “physical activities” OR “physically active” OR “physical exercise” OR exercise) AND (counselling OR counseling OR prescribing OR prescription OR advise OR advice OR educat*)
Platform	(eHealth OR “electronic health” OR computer OR computer-based OR mobile OR device OR phone OR smartphone OR “mobile phone” OR “cell phone” OR mHealth OR “mobile health” OR app OR application OR web OR website OR web-based OR digital OR “digital health”)
Setting	(“primary care” OR “primary health care” OR “primary healthcare” OR “family practice” OR “family medicine” OR “general practice” OR “general practitioner” OR GP)

### Study selection

After duplicate removal, two authors (AW and TT) independently screened titles and abstract. Disagreement about the title and abstract screening was reviewed by the third author (SW) and resolved through consensus. Subsequently, an author (AW) performed the full text review and included the eligible articles. Relevant articles were identified through reference lists and included as additional articles for reviews. The scoping review focused on usability and utility of electronic-based systems for PA counselling in PHC. The inclusion criteria were research or original articles with any study designs conducted in PHC settings and published in peer-reviewed journals. The exclusion criteria were studies conducted in paediatric populations and patients with specific diseases who required specialised care (e.g. cancers, chronic obstructive pulmonary disease, mental disorders). Review articles (i.e. systematic, scoping, narrative reviews), expert opinion excerpts, protocol articles, and trial registers were excluded. The included articles were discussed among the authors prior to data extraction and synthesis.

### Data extraction

One author (AW) performed data extraction using the extraction form developed by the authors. Information from each eligible study included article title, name of first author, year of publication, country of study, study design, participant and setting, type of technology used, counselling domain, variable measurement, and outcomes. Another author (TT or SW) cross-checked the complete data extraction of each study.

### Methodological quality assessment

Two authors (AW and SW) independently assessed the methodological quality of the included studies by using the Mixed Methods Appraisal Tool (MMAT) – Version 2018 [25, 26]. The MMAT is a critical appraisal tool designed for reviews that included mixed types of studies [25]. Each included article was appraised by two screening questions. If the article passed the screening questions, the methodological quality criteria would be applied. The MMAT categorises study designs into five types: (i) qualitative; (ii) quantitative randomised controlled trials; (iii) quantitative nonrandomized; (iv) quantitative descriptive; and (v) mixed methods. Within each type, five items were assessed by dichotomous questions (yes/no or cannot tell). Therefore, the scoring system was 0–5. For mixed methods studies, the assessment covered three types of study designs: (v) mixed methods; (i) qualitative; and either type of quantitative (ii) or (iii) or (iv), consequently, the scores were 0–15 [25].

### Data synthesis

Two authors (AW and either TT or SW) independently performed data synthesis based on the data extraction. The scope of usability included easiness and pleasantness of user interfaces of eHealth for PA counselling [27]. The utility referred to a state that the eHealth for PA counselling provided user needs [27]. Based on the initial review of the included articles, the authors found that the outcomes of usability and utility varied across studies. For example, some studies asked a global rating scale (e.g. overall satisfaction) to rate the usability, while others divided usability into several aspects (e.g. easiness, appearance, support). To summarise the outcomes of each article, the key outcomes are presented in Additional file 2.

In addition, the authors identified three categories of the outcomes to quantify the variation among articles: (i) effective outcomes were noticeably addressed (or most aspects were rated ≥66.66%); (ii) controversial or neutral outcomes were addressed (or most aspects were rated between 33.33 and 66.66%); and (iii) ineffective outcomes were noticeably addressed (or most aspects were rated ≤33.33%). For example, an article presented ‘an average overall satisfaction greater than 3.3 out of 5’ or ‘more than 66.66% of participants satisfied’, it would be considered ‘effective’. If an article reported several aspects of usability or utility, the authors would consider each aspect and decided whether the majority of aspects scored: (i) ≥ 66.66% - effective; (ii) between 33.33 and 66.66% - controversial; or (iii) ≤ 33.33% - ineffective. A third author involved in consensus to resolve any ambiguous results in data synthesis.

## Results

### Summary of search results and study selection

**Fig. 1:**
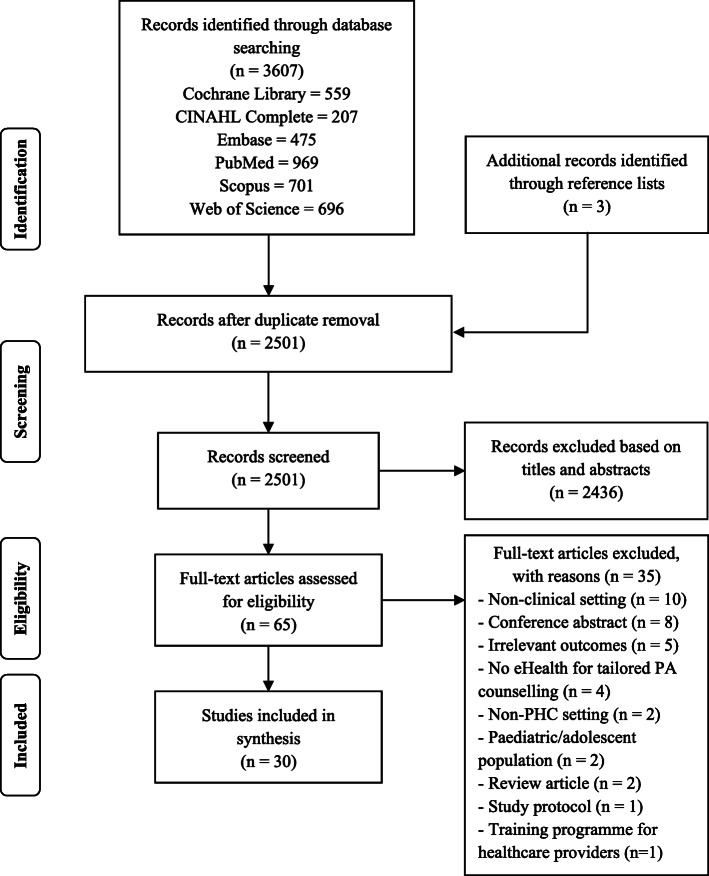
PRISMA flow diagram of search process and results

The initial search in six databases obtained 3607 articles, and 1109 duplicates were removed. Of 2501 articles, 2436 articles were excluded based on reading titles and abstracts by two independent authors. A total of 65 full-text articles were read, and 35 articles were excluded. The number of studies included in data synthesis was 30. Figure 1 presents the PRISMA flow diagram.

### Methodological quality assessment

Of 30 articles, four articles were rated 100% of the items (5/5 items) related to methodological quality [28–31]. Four mixed methods studies were rated ranging from 66% (10/15 items) to over 80% (13/15 items) [18, 32–34]. The rest of the articles were rated 60% (3/5 items, *n* = 10) [20, 35–43] or 80% (4/5 items, *n* = 12) [21, 44–54]. The MMAT scores are presented in Table 2 and Additional file 1.

**Table 2:** Summary of the outcomes of the physical activity domain

Authors, year of publication	MMAT score	Counselling domain	eHealth used	Usability^a^		Utility^a^	
				Patients	Providers	Patients	Providers
Prochaska et al. [44], 2000	4/5	PA and nutrition	PC and internet (web-based programme)	+		+	+
Calfas et al. [45], 2002	4/5	PA and nutrition	Computer programme		+	+	+
Pinto et al. [35], 2002	3/5	PA	TLC used computer technology	+		+	
Anhøj et al. [36], 2004	3/5	PA and diet	Internet based programme	–	–	–	–
Sciamanna et al. [33], 2004	11/15^b^	PA and smoking	Computer-tailored health communication		–		–
Carlfjord et al. [46], 2009	4/5	PA and alcohol	Computer-based lifestyle intervention	+		+	
Carlfjord et al. [47], 2010	4/5	PA and alcohol	Computer-based lifestyle intervention		±		+
Carroll et al. [37], 2010	3/5	PA	Computerised tailored report			±	
Becker et al. [18], 2011	10/15^b^	PA	Computer-based counselling system	±		±	
Christian et al. [29], 2011	5/5	PA and diet	Computer support programme			+	
Leijon et al. [48], 2011	4/5	PA and alcohol	Electronic screening and brief intervention			+	
De Coker et al. [38], 2012	3/5	PA	Computer-tailored website	+		–	
Parekh et al. [49], 2012	4/5	PA, smoking, alcohol, and diet	Personalised computer-tailored feedback			–	
Casey et al. [28], 2014	5/5	PA	Smartphone app	±		+	
Glynn et al. [21], 2014	4/5	PA	Smartphone app			+	
Parekh et al. [50], 2014	4/5	PA, smoking, alcohol, and diet	Personalised computer-tailored feedback			–	
Verwey et al. [39], 2014	3/5	PA	Iterative user-centered mobile technology (smartphone, internet app, pedometer)	+	±	+	+
van der Weegen et al. [31], 2015	5/5	PA	Three-dimensional activity monitor, mobile phone app, and web app			+	
Choo et al. [51], 2016	4/5	PA and weight reduction	Mobile app linked with an accelerometer	±		+	
Diaz et al. [52], 2016	4/5	PA, nutrition, weight, smoking, and alcohol	Tablet-based risk assessment programme	+		–	
Mann et al. [53], 2016	4/5	PA and diet	Shared goal-setting tool embedded in EMR			+	
Recio-Rodriguez et al. [40], 2016	3/5	PA and diet	Mobile phone app			±	
Verwey et al. [34], 2016	10/15^b^	PA	Iterative user-centered mobile technology (smartphone, internet app, pedometer)	+	+		
Walters et al. [41], 2017	3/5	Health and social risks (included PA)	Tailored computer-aided health and social risk appraisal system				±
Degroote et al. [20], 2018	3/5	PA and nutrition	Website			+	
Garcia-Ortiz et al. [42], 2018	3/5	PA and diet	Smartphone app			–	
Glynn et al. [30], 2018	5/5	PA	Smartphone app	±	±	+	+
Poppe et al. [32], 2018	13/15^b^	PA and nutrition	Online programme		±	–	±
Abu-Saad et al. [54], 2019	4/5	PA and diet	Computer software	+		–	
Gill et al. [43], 2019	3/5	PA and diet	Customised health technology tools			+	

*App* application, *EMR* electronic medical record, *MMAT* Mixed Methods Appraisal Tool, *PA* physical activity, *PC* personal computer, *TLC* telephone linked communication

^a^+ = effective outcomes were noticeably addressed (or most aspects were rated ≥66.66%). ± = controversial or neutral outcomes were addressed (or most aspects were rated between 33.33 and 66.66%). - = ineffective outcomes were noticeably addressed (or most aspects were rated ≤33.33%). The blanks refer to no outcome available

^b^mixed methods study

### Counselling domains, eHealth used, and counselling processes

Ten out of 30 articles focused on a stand-alone PA domain [18, 21, 28, 30, 31, 34, 35, 37–39]. The rest of the articles embedded other components of health behaviours or counselling domains. Diet or nutrition was the most common element combined with PA. Some eHealth tools provided counselling about smoking, alcohol consumption, weight control, or the integration of multiple health behaviours (Additional file 2).

Based on the extraction of the included articles, the majority of eHealth PA counselling tools were computer-based technologies. An article published in 2002 presented the usage of telephone linked communication based on computer technology [45]. Of 17 articles published from 2014 to 2019, more than half (*n* = 10) obviously presented the use of mobile technologies (e.g. smartphone, tablet) [21, 28, 30, 31, 34, 39, 40, 42, 51, 52], while some programmes were potential to access by mobile devices (Table 2 and Additional file 2) [20, 32].

Several eHealth tools presented in the included articles were published in combination with other articles. The two articles published in 2000 and 2002 illustrated the Patient-centred Assessment and Counseling for Exercise plus Nutrition (PACE+) [44, 45]. A tool developed in Sweden had been published in 2009 to 2011 [46–48]. Parekh et al. investigated the short-term and long-term effects of eHealth at 3 months and 12 months [49, 50]. An intervention, SMART MOVE, conducted in Ireland were published in different occasions, including qualitative studies and a randomised controlled trial [21, 28, 30]. The research group in the Netherlands produced ‘It’s LiFe!’ as an intervention for promoting PA in PHC [31, 34, 39]. The Spanish team introduced a randomised controlled trial to investigate the short- and long-term effects on utilisation of a mobile phone app [40, 42]. The online programme, MyPlan 1.0, developed in Belgium was presented in two articles (Table 2 and Additional file 2) [20, 32].

With regard to the counselling processes, eHealth technologies were diversely utilised as PA counselling and promoting tools (Additional file 2). Some tools were designed for patients without any interactions with providers [38, 49, 50]. While some eHealth tools were used in a combination of multiple face-to-face consultations [31, 34, 39]. Some articles presented the use of a mobile app, as part of the intervention, to provide PA tracking and tailored feedback [21, 28, 30].

### Usability and utility of eHealth for physical activity counselling

The majority of articles highlighted the usability and/or utility of eHealth for PA counselling for patients or participants rather than PHC providers. In terms of studies investigated patients’ outcomes, most articles (86.67%, *n* = 26/30) investigated the utility of eHealth, while less than half (43.33%, *n* = 13/30) presented the usability. Providers’ outcomes were presented as usability (26.67%, *n* = 8/30) and utility (30.00%, *n* = 9/30). The summary of the extraction and findings are shown in Table 2 and Additional file 2.

#### Usability

Patients’ usability of eHealth was effective or positive in most articles (61.54%, *n =* 8/13) [34, 35, 38, 39, 44, 46, 52, 54]. Four articles (30.77%) showed both satisfaction (e.g. easiness) and dissatisfaction in diverse factors (e.g. time consumption, phone battery consumption, technological issues) [18, 28, 30, 51]. An article (7.69%) published in 2004 noted that the internet based programme was complicated for patients [36].

Of eight articles investigated providers’ usability, two articles (25.00%) reported effective outcomes (e.g. high satisfaction) [34, 45]. Four articles (50.00%) reported controversial outcomes (e.g. easiness vs technical issues) [30, 32, 39, 47]. Two articles (25.00%) stated ineffective usability among PHC providers due to inexperienced staff, complications of the programme, and technical problems [33, 36].

#### Utility

A total of 26 articles reported utility aspects among patients. Fifteen articles (57.69%) showed effective outcomes (e.g. improvement of PA participation, changes in knowledge, attitude, and goal setting) [20, 21, 28–31, 35, 39, 43–46, 48, 51, 53]. Three articles (11.54%) reported both significant and insignificant outcomes of different variables [18, 37, 40]. Eight articles (30.77%) illustrated ineffective outcomes (e.g. no significant increase in PA levels) [32, 36, 38, 42, 49, 50, 52, 54].

Among nine articles indicated providers’ utility, five articles (55.56%) reported effective outcomes (e.g. usefulness of eHealth) [30, 39, 44, 45, 47]. Two articles (22.22%) presented the feasibility of eHealth for PA counselling in PHC, however some barriers to implement the eHealth were addressed such as intervention costs [32, 41]. The rest of the artilces (22.22%, *n* = 2) stated unfavourable outcomes such as technical errors of the programme, and time consuming [33, 36].

## Discussion

### Summary

This scoping review identified usability and utility of eHealth for tailored PA counselling in PHC. Thirty articles were included for analysis. The eHealth tools had a wide range of counselling domains as a stand-alone PA domain and multiple health behaviours. Computer-based technologies represented a dominant eHealth used for PA counselling and promotion in PHC. Mobile technologies (e.g. smartphone, tablet) had been favourable methods since 2014. The eHealth technologies were applied in different approaches with or without patient-provider interactions. The included articles presented mixed findings of usability and utility of eHealth for PA counselling among patients and providers in PHC settings.

Patients’ usability of eHealth was effective or positive in most articles (61.54%), controversial (30.77%), and ineffective (7.69%). In terms of providers’ usability, relevant articles presented effective (25.00%), controversial (50.00%), and ineffective (25.00%). Technical problems and the complexity of the programmes were highlighted as barriers to usability. The majority of articles reported effective utility, however, several articles stated unfavourable outcomes. According to the utility aspects among patients, the inconsistent findings were reported: effective (57.69%); controversial (11.54%); and ineffective (30.77%). Provider’s utility results were effective (55.56%), controversial (22.22%), and ineffective (22.22%).

### Strengths and limitations

There were some strengths of this scoping review. First, the systematic search was performed through six databases, which covered the major and specialised databases for systematic reviews [55]. Second, the scoping review focused on eHealth in PHC settings, which delivered a variety of services [56]. The specific focus could determine particular characteristics of eHealth for PA counselling in PHC settings. Third, the inclusion criteria did not limit study designs. Therefore, this increased yields on articles included and a wide range of findings.

Three major limitations were addressed. First, the scoping review analysed the findings of each article qualitatively. However, the authors considered the findings based on the consensus. Second, a meta-analysis was not performed for quantitative studies. The authors attempted to summarise the outcomes of each article by identifying the criteria to quantify the findings. Third, a diversity of outcome measurements and study designs affected the ability to identify the exact outcomes regarding usability and utility. This revealed the characteristics of scoping reviews, which mainly identified key characteristics related to the concept and knowledge gaps rather than investigating conflict results [57].

### Comparison with existing literature

According to the findings of this scoping review, a variety of eHealth interventions were adopted for PA counselling. A review published in 2007 focused on the effects of eHealth interventions for PA and dietary behaviour change rather than the usability and utility [23]. A systematic review and meta-analysis of Kwan et al. demonstrated a diversity of eHealth strategies for promoting PA in older people and positive effects on time spent on PA, energy expenditure, and step counts [22]. Several eHealth technologies in this scoping review were in line with Kwan et al.’s findings (e.g. automated advice, tele-counselling, PA auto-tracking feedback), however, videogame interventions were not identified in this scoping review as previously mentioned in a systematic review [22].

eHealth interventions were also widely utilised and systematically reviewed for several health behaviours and conditions, such as smoking cessation, overweight, and obesity, which were common in PHC [58–60]. Aforementioned systematic reviews manifested the divergent findings. Nevertheless, their findings may shed light on effective approaches in PHC settings. Tailored interventions were more effective in supporting weight reduction and smoking cessation [58, 60]. A systematic review of Hutchesson et al. presented that 40% of the included studies used more than one type of technologies [59], which were in line with some articles in our scoping review [31, 34, 39]. Multiple options used may help overwhelm barriers and improve the potency of healthcare-based interventions [61].

### Implications for research and practice

The authors highlight two potential implications for future research. First, in this scoping review, reporting structures and outcome measurements are different among studies with various study designs. Therefore, it is challenging to synthesise and interpret the applicability and validity of each study. The standard reporting guidelines may be useful for eHealth studies to provide components for assessing the applicability and validity of the studies. For example, Baker et al. recommended the CONSORT Selected Criteria Adapted for eHealth randomised controlled trials [62]. The CONSORT-EHEALTH (Consolidated Standards of Reporting Trials of Electronic and Mobile HEalth Applications and onLine TeleHealth) is also recommended for reporting eHealth randomised controlled trials [63]. Reporting guidelines for other study designs should be developed to enhance the quality and transparency of eHealth research. Second, future research should focus on the implementation of eHealth for PA counselling and promotion in PHC. Implementation research helps identify implementation challenges in real-world settings [64]. In addition, implementation research offers the understanding of indicators that contribute to the successful implementation, such as acceptability, adoption, appropriateness, cost, coverage, feasibility, fidelity, and sustainability [65].

In PHC practices, eHealth technologies are potential to support PA counselling. However, challenges of PA counselling and the use of eHealth in PHC are addressed. Time constraint is a key barrier to PA counselling in PHC [14, 15]. This factor varies in healthcare settings. For example, a primary care physician consultation time could range from 48 s to 22.5 min [66]. An eHealth intervention should be designed for a specific setting. As a result, an eHealth intervention that consumes merely few minutes may suit a short consultation time space. Ones that required a longer period to participate in the eHealth technologies should be assigned in the waiting rooms or patient’s homes. Moreover, recent technologies (e.g. mobile apps) can provide some clinical tasks with less support by PHC providers, especially, for health promoting tasks [67]. This may help optimise resources in PHC settings. In addition, technical issues are noted in this scoping review. A user-friendly tool for patients and providers should be considered to overcome the technical difficulties. Implementing an eHealth system should take into account of several factors such as appropriateness (e.g. complexity, adaptability, compatibility with existing systems and practices, cost, safety, evidence-based components, quality), provision of training and education, and key stakeholders [68, 69].

## Conclusions

This scoping review found mixed findings in terms of usability and utility of eHealth for PA counselling among patients and providers in PHC settings. Barriers to eHealth usability (e.g. technical issues) should be considered and adapted to a particular PHC setting. The use of eHealth interventions for promoting PA among patients should be pragmatic in order to optimise resources.

## Supplementary Information

**Additional file 1.** Summary of methodological assessment

**Additional file 2.** Summary of the included studies

## Data Availability

All data analysed during this study are included in this published article and its additional files.

## Abbreviations

eHealth: Electronic health
PA: Physical activity
PHC: Primary health care
