# Supplementary material for: Usability and utility of eHealth for physical activity counselling in primary health care: a scoping review
Source: BMC Fam Pract. 2020 Nov 6;21:229. doi: 10.1186/s12875-020-01304-9 (PMC7648312; doi:10.1186/s12875-020-01304-9)
Supplement: Supplementary file 1 — Additional file 1. Summary of methodological assessment [file 12875_2020_1304_MOESM1_ESM.docx]

**Additional File 1** Summary of methodological assessment

**Article information**

**Article title:** PACE+ Interactive Communication Technology for Behavior Change in Clinical Settings

**First author name:** Judith J. Prochaska

**Year of publication:** 2000

**Country of study:** USA

**Methodological quality assessment (Mixed Methods Appraisal Tool (MMAT), version 2018)**

| **Category of study designs** | **Methodological quality criteria** | **Response** | | | |
| --- | --- | --- | --- | --- | --- |
|  |  | **Yes** | **No** | **Can’t tell** | **Comments** |
| Screening questions (for all types) | S1. Are there clear research questions? | ✓ |  |  |  |
|  | S2. Do the collected data allow to address the research questions? | ✓ |  |  |  |
|  | *Further appraisal may not be feasible or appropriate when the answer is ‘No’ or ‘Can’t tell’ to one or both screening questions.* | | | | |
| 4. Quantitative  descriptive | 4.1. Is the sampling strategy relevant to address the research question? | ✓ |  |  | contacting patients  before a scheduled appointment and by  approaching patients on site.  67% of adolescents and 69% of adults accepted to participate in the study. |
|  | 4.2. Is the sample representative of the target population? | ✓ |  |  |  |
|  | 4.3. Are the measurements appropriate? | ✓ |  |  |  |
|  | 4.4. Is the risk of nonresponse bias low? |  |  | ✓ |  |
|  | 4.5. Is the statistical analysis appropriate to answer the research question? | ✓ |  |  |  |

**Article information**

**Article title:** Preliminary Evaluation of a Multicomponent Program for Nutrition and Physical Activity Change in Primary Care: PACE+ for Adults

**First author name:** Karen J. Calfas

**Year of publication:** 2002

**Country of study:** USA

**Methodological quality assessment (Mixed Methods Appraisal Tool (MMAT), version 2018)**

| **Category of study designs** | **Methodological quality criteria** | **Response** | | | |
| --- | --- | --- | --- | --- | --- |
|  |  | **Yes** | **No** | **Can’t tell** | **Comments** |
| Screening questions (for all types) | S1. Are there clear research questions? | ✓ |  |  |  |
|  | S2. Do the collected data allow to address the research questions? | ✓ |  |  |  |
|  | *Further appraisal may not be feasible or appropriate when the answer is ‘No’ or ‘Can’t tell’ to one or both screening questions.* | | | | |
| 3. Quantitative nonrandomized | 3.1. Are the participants representative of the target population? | ✓ |  |  |  |
|  | 3.2. Are measurements appropriate regarding both the outcome and intervention (or exposure)? | ✓ |  |  |  |
|  | 3.3. Are there complete outcome data? | ✓ |  |  |  |
|  | 3.4. Are the confounders accounted for in the design and analysis? |  | ✓ |  |  |
|  | 3.5. During the study period, is the intervention administered (or exposure occurred) as intended? | ✓ |  |  |  |

**Article information**

**Article title:** Effects of a computer-based, telephone-counseling system on physical activity

**First author name:** Bernardine M. Pinto

**Year of publication:** 2002

**Country of study:** USA

**Methodological quality assessment (Mixed Methods Appraisal Tool (MMAT), version 2018)**

| **Category of study designs** | **Methodological quality criteria** | **Response** | | | |
| --- | --- | --- | --- | --- | --- |
|  |  | **Yes** | **No** | **Can’t tell** | **Comments** |
| Screening questions (for all types) | S1. Are there clear research questions? | **✓** |  |  |  |
|  | S2. Do the collected data allow to address the research questions? | **✓** |  |  |  |
|  | *Further appraisal may not be feasible or appropriate when the answer is ‘No’ or ‘Can’t tell’ to one or both screening questions.* | | | | |
| 2. Quantitative  randomized controlled  trials | 2.1. Is randomization appropriately performed? | **✓** |  |  | Some subjects (33%) did not call the system at all,  and there was a significant drop in the number of users  over 6 months |
|  | 2.2. Are the groups comparable at baseline? | **✓** |  |  |  |
|  | 2.3. Are there complete outcome data? | **✓** |  |  |  |
|  | 2.4. Are outcome assessors blinded to the intervention provided? |  | **✓** |  |  |
|  | 2.5 Did the participants adhere to the assigned intervention? |  | **✓** |  |  |

**Article information**

**Article title:** Using the internet for lifestyle changes in diet and physical activity: A feasibility study

**First author name:** Jacob Anhøj

**Year of publication:** 2004

**Country of study:** Denmark

**Methodological quality assessment (Mixed Methods Appraisal Tool (MMAT), version 2018)**

| **Category of study designs** | **Methodological quality criteria** | **Response** | | | |
| --- | --- | --- | --- | --- | --- |
|  |  | **Yes** | **No** | **Can’t tell** | **Comments** |
| Screening questions (for all types) | S1. Are there clear research questions? | **✓** |  |  |  |
|  | S2. Do the collected data allow to address the research questions? | **✓** |  |  |  |
|  | *Further appraisal may not be feasible or appropriate when the answer is ‘No’ or ‘Can’t tell’ to one or both screening questions.* | | | | |
| 1. Qualitative | 1.1. Is the qualitative approach appropriate to answer the research question? | **✓** |  |  | Qualitative processes are described briefly |
|  | 1.2. Are the qualitative data collection methods adequate to address the research question? | **✓** |  |  |  |
|  | 1.3. Are the findings adequately derived from the data? |  |  | **✓** |  |
|  | 1.4. Is the interpretation of results sufficiently substantiated by data? | **✓** |  |  |  |
|  | 1.5. Is there coherence between qualitative data sources, collection, analysis and interpretation? |  |  | **✓** |  |

**Article information**

**Article title:** Feasibility of incorporating computer-tailored health behaviour communications in primary care settings

**First author name:** Christopher N Sciamanna

**Year of publication:** 2004

**Country of study:** USA

**Methodological quality assessment (Mixed Methods Appraisal Tool (MMAT), version 2018)**

| **Category of study designs** | **Methodological quality criteria** | **Response** | | | |
| --- | --- | --- | --- | --- | --- |
|  |  | **Yes** | **No** | **Can’t tell** | **Comments** |
| Screening questions (for all types) | S1. Are there clear research questions? | **✓** |  |  |  |
|  | S2. Do the collected data allow to address the research questions? | **✓** |  |  |  |
|  | *Further appraisal may not be feasible or appropriate when the answer is ‘No’ or ‘Can’t tell’ to one or both screening questions.* | | | | |
| 1. Qualitative | 1.1. Is the qualitative approach appropriate to answer the research question? | **✓** |  |  |  |
|  | 1.2. Are the qualitative data collection methods adequate to address the research question? | **✓** |  |  |  |
|  | 1.3. Are the findings adequately derived from the data? |  | **✓** |  |  |
|  | 1.4. Is the interpretation of results sufficiently substantiated by data? | **✓** |  |  |  |
|  | 1.5. Is there coherence between qualitative data sources, collection, analysis and interpretation? |  | **✓** |  |  |
| 4. Quantitative  descriptive | 4.1. Is the sampling strategy relevant to address the research question? | **✓** |  |  |  |
|  | 4.2. Is the sample representative of the target population? | **✓** |  |  |  |
|  | 4.3. Are the measurements appropriate? | **✓** |  |  |  |
|  | 4.4. Is the risk of nonresponse bias low? | **✓** |  |  |  |
|  | 4.5. Is the statistical analysis appropriate to answer the research question? | **✓** |  |  |  |
| 5. Mixed methods | 5.1. Is there an adequate rationale for using a mixed methods design to address the research question? | **✓** |  |  |  |
|  | 5.2. Are the different components of the study effectively integrated to answer the research question? | **✓** |  |  |  |
|  | 5.3. Are the outputs of the integration of qualitative and quantitative components adequately interpreted? | **✓** |  |  |  |
|  | 5.4. Are divergences and inconsistencies between quantitative and qualitative results adequately addressed? |  |  | **✓** |  |
|  | 5.5. Do the different components of the study adhere to the quality criteria of each tradition of the methods involved? |  | **✓** |  |  |

**Article information**

**Article title:** Computerized lifestyle intervention in routine primary health care: Evaluation of usage on provider and responder levels

**First author name:** S. Carlfjord

**Year of publication:** 2009

**Country of study:** Sweden

**Methodological quality assessment (Mixed Methods Appraisal Tool (MMAT), version 2018)**

| **Category of study designs** | **Methodological quality criteria** | **Response** | | | |
| --- | --- | --- | --- | --- | --- |
|  |  | **Yes** | **No** | **Can’t tell** | **Comments** |
| Screening questions (for all types) | S1. Are there clear research questions? | ✓ |  |  |  |
|  | S2. Do the collected data allow to address the research questions? | ✓ |  |  |  |
|  | *Further appraisal may not be feasible or appropriate when the answer is ‘No’ or ‘Can’t tell’ to one or both screening questions.* | | | | |
| 4. Quantitative  descriptive | 4.1. Is the sampling strategy relevant to address the research question? | ✓ |  |  | During the 1-year study period, 5202 tests were initiated, of  which 3065 tests (59%) were completed. 38 respondents were excluded due to outliers (>100 standard drinks/week) |
|  | 4.2. Is the sample representative of the target population? | ✓ |  |  |  |
|  | 4.3. Are the measurements appropriate? | ✓ |  |  |  |
|  | 4.4. Is the risk of nonresponse bias low? |  |  | ✓ |  |
|  | 4.5. Is the statistical analysis appropriate to answer the research question? | ✓ |  |  |  |

**Article information**

**Article title:** Staff perspectives on the use of a computer-based concept for lifestyle intervention implemented in primary health care

**First author name:** Siw Carlfjord

**Year of publication:** 2010

**Country of study:** Sweden

**Methodological quality assessment (Mixed Methods Appraisal Tool (MMAT), version 2018)**

| **Category of study designs** | **Methodological quality criteria** | **Response** | | | |
| --- | --- | --- | --- | --- | --- |
|  |  | **Yes** | **No** | **Can’t tell** | **Comments** |
| Screening questions (for all types) | S1. Are there clear research questions? | ✓ |  |  |  |
|  | S2. Do the collected data allow to address the research questions? | ✓ |  |  |  |
|  | *Further appraisal may not be feasible or appropriate when the answer is ‘No’ or ‘Can’t tell’ to one or both screening questions.* | | | | |
| 4. Quantitative  descriptive | 4.1. Is the sampling strategy relevant to address the research question? | ✓ |  |  | 59% response rate |
|  | 4.2. Is the sample representative of the target population? | ✓ |  |  |  |
|  | 4.3. Are the measurements appropriate? | ✓ |  |  |  |
|  | 4.4. Is the risk of nonresponse bias low? |  | ✓ |  |  |
|  | 4.5. Is the statistical analysis appropriate to answer the research question? | ✓ |  |  |  |

**Article information**

**Article title:** Computerized tailored physical activity reports A randomized controlled trial

**First author name:** Jennifer K. Carroll

**Year of publication:** 2010

**Country of study:** USA

**Methodological quality assessment (Mixed Methods Appraisal Tool (MMAT), version 2018)**

| **Category of study designs** | **Methodological quality criteria** | **Response** | | | |
| --- | --- | --- | --- | --- | --- |
|  |  | **Yes** | **No** | **Can’t tell** | **Comments** |
| Screening questions (for all types) | S1. Are there clear research questions? | ✓ |  |  |  |
|  | S2. Do the collected data allow to address the research questions? | ✓ |  |  |  |
|  | *Further appraisal may not be feasible or appropriate when the answer is ‘No’ or ‘Can’t tell’ to one or both screening questions.* | | | | |
| 2. Quantitative  randomized controlled  trials | 2.1. Is randomization appropriately performed? | ✓ |  |  | Gender and age were different at baseline |
|  | 2.2. Are the groups comparable at baseline? |  | ✓ |  |  |
|  | 2.3. Are there complete outcome data? | ✓ |  |  |  |
|  | 2.4. Are outcome assessors blinded to the intervention provided? |  |  | ✓ |  |
|  | 2.5 Did the participants adhere to the assigned intervention? | ✓ |  |  |  |

**Article information**

**Article title:** A new computer-based counselling system for the promotion of physical activity in patients with chronic diseases—Results from a pilot study

**First author name:** Annette Becker

**Year of publication:** 2011

**Country of study:** Germany

**Methodological quality assessment (Mixed Methods Appraisal Tool (MMAT), version 2018)**

| **Category of study designs** | **Methodological quality criteria** | **Response** | | | |
| --- | --- | --- | --- | --- | --- |
|  |  | **Yes** | **No** | **Can’t tell** | **Comments** |
| Screening questions (for all types) | S1. Are there clear research questions? | ✓ |  |  |  |
|  | S2. Do the collected data allow to address the research questions? | ✓ |  |  |  |
|  | *Further appraisal may not be feasible or appropriate when the answer is ‘No’ or ‘Can’t tell’ to one or both screening questions.* | | | | |
| 1. Qualitative | 1.1. Is the qualitative approach appropriate to answer the research question? | ✓ |  |  |  |
|  | 1.2. Are the qualitative data collection methods adequate to address the research question? | ✓ |  |  |  |
|  | 1.3. Are the findings adequately derived from the data? |  |  | ✓ |  |
|  | 1.4. Is the interpretation of results sufficiently substantiated by data? | ✓ |  |  |  |
|  | 1.5. Is there coherence between qualitative data sources, collection, analysis and interpretation? | ✓ |  |  |  |
| 3. Quantitative nonrandomized | 3.1. Are the participants representative of the target population? |  |  | ✓ | Convenient sampling, dropout rate = 22% |
|  | 3.2. Are measurements appropriate regarding both the outcome and intervention (or exposure)? | ✓ |  |  |  |
|  | 3.3. Are there complete outcome data? |  | ✓ |  |  |
|  | 3.4. Are the confounders accounted for in the design and analysis? | ✓ |  |  |  |
|  | 3.5. During the study period, is the intervention administered (or exposure occurred) as intended? | ✓ |  |  |  |
| 5. Mixed methods | 5.1. Is there an adequate rationale for using a mixed methods design to address the research question? | ✓ |  |  |  |
|  | 5.2. Are the different components of the study effectively integrated to answer the research question? | ✓ |  |  |  |
|  | 5.3. Are the outputs of the integration of qualitative and quantitative components adequately interpreted? | ✓ |  |  |  |
|  | 5.4. Are divergences and inconsistencies between quantitative and qualitative results adequately addressed? |  |  | ✓ |  |
|  | 5.5. Do the different components of the study adhere to the quality criteria of each tradition of the methods involved? |  |  | ✓ |  |

**Article information**

**Article title:** A computer support program that helps clinicians provide patients with metabolic syndrome tailored counseling to promote weight loss

**First author name:** James G. Christian

**Year of publication:** 2011

**Country of study:** USA

**Methodological quality assessment (Mixed Methods Appraisal Tool (MMAT), version 2018)**

| **Category of study designs** | **Methodological quality criteria** | **Response** | | | |
| --- | --- | --- | --- | --- | --- |
|  |  | **Yes** | **No** | **Can’t tell** | **Comments** |
| Screening questions (for all types) | S1. Are there clear research questions? | ✓ |  |  |  |
|  | S2. Do the collected data allow to address the research questions? | ✓ |  |  |  |
|  | *Further appraisal may not be feasible or appropriate when the answer is ‘No’ or ‘Can’t tell’ to one or both screening questions.* | | | | |
| 3. Quantitative nonrandomized | 3.1. Are the participants representative of the target population? | ✓ |  |  |  |
|  | 3.2. Are measurements appropriate regarding both the outcome and intervention (or exposure)? | ✓ |  |  |  |
|  | 3.3. Are there complete outcome data? | ✓ |  |  |  |
|  | 3.4. Are the confounders accounted for in the design and analysis? | ✓ |  |  |  |
|  | 3.5. During the study period, is the intervention administered (or exposure occurred) as intended? | ✓ |  |  |  |

**Article information**

**Article title:** Improvement of physical activity by a kiosk-based electronic screening and brief intervention in routine primary health care: Patient-initiated versus staff-referred

**First author name:** Matti Leijon

**Year of publication:** 2011

**Country of study:** Sweden

**Methodological quality assessment (Mixed Methods Appraisal Tool (MMAT), version 2018)**

| **Category of study designs** | **Methodological quality criteria** | **Response** | | | |
| --- | --- | --- | --- | --- | --- |
|  |  | **Yes** | **No** | **Can’t tell** | **Comments** |
| Screening questions (for all types) | S1. Are there clear research questions? | ✓ |  |  |  |
|  | S2. Do the collected data allow to address the research questions? | ✓ |  |  |  |
|  | *Further appraisal may not be feasible or appropriate when the answer is ‘No’ or ‘Can’t tell’ to one or both screening questions.* | | | | |
| 3. Quantitative nonrandomized | 3.1. Are the participants representative of the target population? | ✓ |  |  |  |
|  | 3.2. Are measurements appropriate regarding both the outcome and intervention (or exposure)? | ✓ |  |  |  |
|  | 3.3. Are there complete outcome data? |  | ✓ |  |  |
|  | 3.4. Are the confounders accounted for in the design and analysis? | ✓ |  |  |  |
|  | 3.5. During the study period, is the intervention administered (or exposure occurred) as intended? | ✓ |  |  |  |

**Article information**

**Article title:** Web-based, computer-tailored, pedometer-based physical activity advice: Development, dissemination through general practice, acceptability, and preliminary efficacy in a randomized controlled trial

**First author name:** Katrien De Cocker

**Year of publication:** 2012

**Country of study:** Belgium

**Methodological quality assessment (Mixed Methods Appraisal Tool (MMAT), version 2018)**

| **Category of study designs** | **Methodological quality criteria** | **Response** | | | |
| --- | --- | --- | --- | --- | --- |
|  |  | **Yes** | **No** | **Can’t tell** | **Comments** |
| Screening questions (for all types) | S1. Are there clear research questions? | ✓ |  |  |  |
|  | S2. Do the collected data allow to address the research questions? | ✓ |  |  |  |
|  | *Further appraisal may not be feasible or appropriate when the answer is ‘No’ or ‘Can’t tell’ to one or both screening questions.* | | | | |
| 2. Quantitative  randomized controlled  trials | 2.1. Is randomization appropriately performed? |  |  | ✓ | n = 92, 23 dropouts  (intention-to-treat analysis) |
|  | 2.2. Are the groups comparable at baseline? | ✓ |  |  |  |
|  | 2.3. Are there complete outcome data? | ✓ |  |  |  |
|  | 2.4. Are outcome assessors blinded to the intervention provided? |  |  | ✓ |  |
|  | 2.5 Did the participants adhere to the assigned intervention? | ✓ |  |  |  |

**Article information**

**Article title:** Improving diet, physical activity and other lifestyle behaviours using computer-tailored advice in general practice: a randomized controlled trial

**First author name:** Sanjoti Parekh

**Year of publication:** 2012

**Country of study:** Australia

**Methodological quality assessment (Mixed Methods Appraisal Tool (MMAT), version 2018)**

| **Category of study designs** | **Methodological quality criteria** | **Response** | | | |
| --- | --- | --- | --- | --- | --- |
|  |  | **Yes** | **No** | **Can’t tell** | **Comments** |
| Screening questions (for all types) | S1. Are there clear research questions? | ✓ |  |  |  |
|  | S2. Do the collected data allow to address the research questions? | ✓ |  |  |  |
|  | *Further appraisal may not be feasible or appropriate when the answer is ‘No’ or ‘Can’t tell’ to one or both screening questions.* | | | | |
| 2. Quantitative  randomized controlled  trials | 2.1. Is randomization appropriately performed? | ✓ |  |  | 76% completed the survey, intention-to-treat analysis |
|  | 2.2. Are the groups comparable at baseline? | ✓ |  |  |  |
|  | 2.3. Are there complete outcome data? | ✓ |  |  |  |
|  | 2.4. Are outcome assessors blinded to the intervention provided? |  | ✓ |  |  |
|  | 2.5 Did the participants adhere to the assigned intervention? | ✓ |  |  |  |

**Article information**

**Article title:** Patients’ experiences of using a smartphone application to increase physical activity: the SMART MOVE qualitative study in primary care

**First author name:** Monica Casey

**Year of publication:** 2014

**Country of study:** Ireland

**Methodological quality assessment (Mixed Methods Appraisal Tool (MMAT), version 2018)**

| **Category of study designs** | **Methodological quality criteria** | **Response** | | | |
| --- | --- | --- | --- | --- | --- |
|  |  | **Yes** | **No** | **Can’t tell** | **Comments** |
| Screening questions (for all types) | S1. Are there clear research questions? | ✓ |  |  |  |
|  | S2. Do the collected data allow to address the research questions? | ✓ |  |  |  |
|  | *Further appraisal may not be feasible or appropriate when the answer is ‘No’ or ‘Can’t tell’ to one or both screening questions.* | | | | |
| 1. Qualitative | 1.1. Is the qualitative approach appropriate to answer the research question? | ✓ |  |  |  |
|  | 1.2. Are the qualitative data collection methods adequate to address the research question? | ✓ |  |  |  |
|  | 1.3. Are the findings adequately derived from the data? | ✓ |  |  |  |
|  | 1.4. Is the interpretation of results sufficiently substantiated by data? | ✓ |  |  |  |
|  | 1.5. Is there coherence between qualitative data sources, collection, analysis and interpretation? | ✓ |  |  |  |

**Article information**

**Article title:** Effectiveness of a smartphone application to promote physical activity in primary care: the SMART MOVE randomised controlled trial

**First author name:** Liam G Glynn

**Year of publication:** 2014

**Country of study:** Ireland

**Methodological quality assessment (Mixed Methods Appraisal Tool (MMAT), version 2018)**

| **Category of study designs** | **Methodological quality criteria** | **Response** | | | |
| --- | --- | --- | --- | --- | --- |
|  |  | **Yes** | **No** | **Can’t tell** | **Comments** |
| Screening questions (for all types) | S1. Are there clear research questions? | ✓ |  |  |  |
|  | S2. Do the collected data allow to address the research questions? | ✓ |  |  |  |
|  | *Further appraisal may not be feasible or appropriate when the answer is ‘No’ or ‘Can’t tell’ to one or both screening questions.* | | | | |
| 2. Quantitative  randomized controlled  trials | 2.1. Is randomization appropriately performed? | ✓ |  |  |  |
|  | 2.2. Are the groups comparable at baseline? | ✓ |  |  |  |
|  | 2.3. Are there complete outcome data? | ✓ |  |  |  |
|  | 2.4. Are outcome assessors blinded to the intervention provided? |  | ✓ |  |  |
|  | 2.5 Did the participants adhere to the assigned intervention? | ✓ |  |  |  |

**Article information**

**Article title:** Randomized controlled trial of a computer-tailored multiple health behaviour intervention in general practice: 12-month follow-up results

**First author name:** Sanjoti Parekh

**Year of publication:** 2014

**Country of study:** Australia

**Methodological quality assessment (Mixed Methods Appraisal Tool (MMAT), version 2018)**

| **Category of study designs** | **Methodological quality criteria** | **Response** | | | |
| --- | --- | --- | --- | --- | --- |
|  |  | **Yes** | **No** | **Can’t tell** | **Comments** |
| Screening questions (for all types) | S1. Are there clear research questions? | ✓ |  |  |  |
|  | S2. Do the collected data allow to address the research questions? | ✓ |  |  |  |
|  | *Further appraisal may not be feasible or appropriate when the answer is ‘No’ or ‘Can’t tell’ to one or both screening questions.* | | | | |
| 2. Quantitative  randomized controlled  trials | 2.1. Is randomization appropriately performed? | ✓ |  |  |  |
|  | 2.2. Are the groups comparable at baseline? | ✓ |  |  |  |
|  | 2.3. Are there complete outcome data? | ✓ |  |  |  |
|  | 2.4. Are outcome assessors blinded to the intervention provided? |  |  | ✓ |  |
|  | 2.5 Did the participants adhere to the assigned intervention? | ✓ |  |  |  |

**Article information**

**Article title:** Technology combined with a counseling protocol to stimulate physical activity of chronically ill patients in primary care

**First author name:** R. Verwey

**Year of publication:** 2014

**Country of study:** Netherlands

**Methodological quality assessment (Mixed Methods Appraisal Tool (MMAT), version 2018)**

| **Category of study designs** | **Methodological quality criteria** | **Response** | | | |
| --- | --- | --- | --- | --- | --- |
|  |  | **Yes** | **No** | **Can’t tell** | **Comments** |
| Screening questions (for all types) | S1. Are there clear research questions? | ✓ |  |  |  |
|  | S2. Do the collected data allow to address the research questions? | ✓ |  |  |  |
|  | *Further appraisal may not be feasible or appropriate when the answer is ‘No’ or ‘Can’t tell’ to one or both screening questions.* | | | | |
| 1. Qualitative | 1.1. Is the qualitative approach appropriate to answer the research question? | ✓ |  |  |  |
|  | 1.2. Are the qualitative data collection methods adequate to address the research question? | ✓ |  |  |  |
|  | 1.3. Are the findings adequately derived from the data? |  |  | ✓ |  |
|  | 1.4. Is the interpretation of results sufficiently substantiated by data? |  |  | ✓ |  |
|  | 1.5. Is there coherence between qualitative data sources, collection, analysis and interpretation? | ✓ |  |  |  |

**Article information**

**Article title:** It's LiFe! mobile and web-based monitoring and feedback tool embedded in primary care increases physical activity: A cluster randomized controlled trial

**First author name:** Sanne van der Weegen

**Year of publication:** 2015

**Country of study:** Netherlands

**Methodological quality assessment (Mixed Methods Appraisal Tool (MMAT), version 2018)**

| **Category of study designs** | **Methodological quality criteria** | **Response** | | | |
| --- | --- | --- | --- | --- | --- |
|  |  | **Yes** | **No** | **Can’t tell** | **Comments** |
| Screening questions (for all types) | S1. Are there clear research questions? | **✓** |  |  |  |
|  | S2. Do the collected data allow to address the research questions? | **✓** |  |  |  |
|  | *Further appraisal may not be feasible or appropriate when the answer is ‘No’ or ‘Can’t tell’ to one or both screening questions.* | | | | |
| 2. Quantitative  randomized controlled  trials | 2.1. Is randomization appropriately performed? | **✓** |  |  |  |
|  | 2.2. Are the groups comparable at baseline? | **✓** |  |  |  |
|  | 2.3. Are there complete outcome data? | **✓** |  |  |  |
|  | 2.4. Are outcome assessors blinded to the intervention provided? | **✓** |  |  |  |
|  | 2.5 Did the participants adhere to the assigned intervention? | **✓** |  |  |  |

**Article information**

**Article title:** Development of a weight loss mobile app linked with an accelerometer for use in the clinic: Usability, acceptability, and early testing of its impact on the patient-doctor relationship

**First author name:** Seryung Choo

**Year of publication:** 2016

**Country of study:** Korea

**Methodological quality assessment (Mixed Methods Appraisal Tool (MMAT), version 2018)**

| **Category of study designs** | **Methodological quality criteria** | **Response** | | | |
| --- | --- | --- | --- | --- | --- |
|  |  | **Yes** | **No** | **Can’t tell** | **Comments** |
| Screening questions (for all types) | S1. Are there clear research questions? | ✓ |  |  |  |
|  | S2. Do the collected data allow to address the research questions? | ✓ |  |  |  |
|  | *Further appraisal may not be feasible or appropriate when the answer is ‘No’ or ‘Can’t tell’ to one or both screening questions.* | | | | |
| 3. Quantitative nonrandomized | 3.1. Are the participants representative of the target population? | ✓ |  |  |  |
|  | 3.2. Are measurements appropriate regarding both the outcome and intervention (or exposure)? | ✓ |  |  |  |
|  | 3.3. Are there complete outcome data? | ✓ |  |  |  |
|  | 3.4. Are the confounders accounted for in the design and analysis? |  |  | ✓ |  |
|  | 3.5. During the study period, is the intervention administered (or exposure occurred) as intended? | ✓ |  |  |  |

**Article information**

**Article title:** Use of a tablet-based risk assessment program to improve health counseling and patient–provider relationships in a federally qualified health center

**First author name:** Vanessa A. Diaz

**Year of publication:** 2016

**Country of study:** USA

**Methodological quality assessment (Mixed Methods Appraisal Tool (MMAT), version 2018)**

| **Category of study designs** | **Methodological quality criteria** | **Response** | | | |
| --- | --- | --- | --- | --- | --- |
|  |  | **Yes** | **No** | **Can’t tell** | **Comments** |
| Screening questions (for all types) | S1. Are there clear research questions? | ✓ |  |  |  |
|  | S2. Do the collected data allow to address the research questions? | ✓ |  |  |  |
|  | *Further appraisal may not be feasible or appropriate when the answer is ‘No’ or ‘Can’t tell’ to one or both screening questions.* | | | | |
| 3. Quantitative nonrandomized | 3.1. Are the participants representative of the target population? | ✓ |  |  | 79.8% follow-up rate at the first week |
|  | 3.2. Are measurements appropriate regarding both the outcome and intervention (or exposure)? | ✓ |  |  |  |
|  | 3.3. Are there complete outcome data? |  | ✓ |  |  |
|  | 3.4. Are the confounders accounted for in the design and analysis? | ✓ |  |  |  |
|  | 3.5. During the study period, is the intervention administered (or exposure occurred) as intended? | ✓ |  |  |  |

**Article information**

**Article title:** A pilot randomized trial of technology-assisted goal setting to improve physical activity among primary care patients with prediabetes

**First author name:** Devin M. Mann

**Year of publication:** 2016

**Country of study:** USA

**Methodological quality assessment (Mixed Methods Appraisal Tool (MMAT), version 2018)**

| **Category of study designs** | **Methodological quality criteria** | **Response** | | | |
| --- | --- | --- | --- | --- | --- |
|  |  | **Yes** | **No** | **Can’t tell** | **Comments** |
| Screening questions (for all types) | S1. Are there clear research questions? | ✓ |  |  |  |
|  | S2. Do the collected data allow to address the research questions? | ✓ |  |  |  |
|  | *Further appraisal may not be feasible or appropriate when the answer is ‘No’ or ‘Can’t tell’ to one or both screening questions.* | | | | |
| 2. Quantitative  randomized controlled  trials | 2.1. Is randomization appropriately performed? | ✓ |  |  |  |
|  | 2.2. Are the groups comparable at baseline? | ✓ |  |  |  |
|  | 2.3. Are there complete outcome data? | ✓ |  |  |  |
|  | 2.4. Are outcome assessors blinded to the intervention provided? |  | ✓ |  |  |
|  | 2.5 Did the participants adhere to the assigned intervention? | ✓ |  |  |  |

**Article information**

**Article title:** Short-term effectiveness of a mobile phone app for increasing physical activity and adherence to the Mediterranean diet in primary care: A randomized controlled trial (EVIDENT II Study)

**First author name:** Jose I Recio-Rodriguez

**Year of publication:** 2016

**Country of study:** Spain

**Methodological quality assessment (Mixed Methods Appraisal Tool (MMAT), version 2018)**

| **Category of study designs** | **Methodological quality criteria** | **Response** | | | |
| --- | --- | --- | --- | --- | --- |
|  |  | **Yes** | **No** | **Can’t tell** | **Comments** |
| Screening questions (for all types) | S1. Are there clear research questions? | ✓ |  |  |  |
|  | S2. Do the collected data allow to address the research questions? | ✓ |  |  |  |
|  | *Further appraisal may not be feasible or appropriate when the answer is ‘No’ or ‘Can’t tell’ to one or both screening questions.* | | | | |
| 2. Quantitative  randomized controlled  trials | 2.1. Is randomization appropriately performed? | ✓ |  |  | 56.8% of participants used the app > 60 days |
|  | 2.2. Are the groups comparable at baseline? | ✓ |  |  |  |
|  | 2.3. Are there complete outcome data? | ✓ |  |  |  |
|  | 2.4. Are outcome assessors blinded to the intervention provided? |  |  | ✓ |  |
|  | 2.5 Did the participants adhere to the assigned intervention? |  | ✓ |  |  |

**Article information**

**Article title:** Process evaluation of physical activity counselling with and without the use of mobile technology: A mixed methods study

**First author name:** R. Verwey

**Year of publication:** 2016

**Country of study:** Netherlands

**Methodological quality assessment (Mixed Methods Appraisal Tool (MMAT), version 2018)**

| **Category of study designs** | **Methodological quality criteria** | **Response** | | | |
| --- | --- | --- | --- | --- | --- |
|  |  | **Yes** | **No** | **Can’t tell** | **Comments** |
| Screening questions (for all types) | S1. Are there clear research questions? | ✓ |  |  |  |
|  | S2. Do the collected data allow to address the research questions? | ✓ |  |  |  |
|  | *Further appraisal may not be feasible or appropriate when the answer is ‘No’ or ‘Can’t tell’ to one or both screening questions.* | | | | |
| 1. Qualitative | 1.1. Is the qualitative approach appropriate to answer the research question? | ✓ |  |  |  |
|  | 1.2. Are the qualitative data collection methods adequate to address the research question? | ✓ |  |  |  |
|  | 1.3. Are the findings adequately derived from the data? |  |  | ✓ |  |
|  | 1.4. Is the interpretation of results sufficiently substantiated by data? |  |  | ✓ |  |
|  | 1.5. Is there coherence between qualitative data sources, collection, analysis and interpretation? | ✓ |  |  |  |
| 2. Quantitative  randomized controlled  trials | 2.1. Is randomization appropriately performed? | ✓ |  |  |  |
|  | 2.2. Are the groups comparable at baseline? | ✓ |  |  |  |
|  | 2.3. Are there complete outcome data? | ✓ |  |  |  |
|  | 2.4. Are outcome assessors blinded to the intervention provided? |  |  | ✓ |  |
|  | 2.5 Did the participants adhere to the assigned intervention? | ✓ |  |  |  |
| 5. Mixed methods | 5.1. Is there an adequate rationale for using a mixed methods design to address the research question? | ✓ |  |  |  |
|  | 5.2. Are the different components of the study effectively integrated to answer the research question? | ✓ |  |  |  |
|  | 5.3. Are the outputs of the integration of qualitative and quantitative components adequately interpreted? |  |  | ✓ |  |
|  | 5.4. Are divergences and inconsistencies between quantitative and qualitative results adequately addressed? | ✓ |  |  |  |
|  | 5.5. Do the different components of the study adhere to the quality criteria of each tradition of the methods involved? |  | ✓ |  |  |

**Article information**

**Article title:** Promoting independence, health and well-being for older people: a feasibility study of computer-aided health and social risk appraisal system in primary care

**First author name:** Kate Walters

**Year of publication:** 2017

**Country of study:** UK

**Methodological quality assessment (Mixed Methods Appraisal Tool (MMAT), version 2018)**

| **Category of study designs** | **Methodological quality criteria** | **Response** | | | |
| --- | --- | --- | --- | --- | --- |
|  |  | **Yes** | **No** | **Can’t tell** | **Comments** |
| Screening questions (for all types) | S1. Are there clear research questions? | ✓ |  |  |  |
|  | S2. Do the collected data allow to address the research questions? | ✓ |  |  |  |
|  | *Further appraisal may not be feasible or appropriate when the answer is ‘No’ or ‘Can’t tell’ to one or both screening questions.* | | | | |
| 4. Quantitative  descriptive | 4.1. Is the sampling strategy relevant to address the research question? | ✓ |  |  | Response rates were low (34%) |
|  | 4.2. Is the sample representative of the target population? |  | ✓ |  |  |
|  | 4.3. Are the measurements appropriate? | ✓ |  |  |  |
|  | 4.4. Is the risk of nonresponse bias low? |  |  | ✓ |  |
|  | 4.5. Is the statistical analysis appropriate to answer the research question? | ✓ |  |  |  |

**Article title:** The effect of the eHealth intervention ‘MyPlan 1.0’ on physical activity in adults who visit general practice: A quasi-experimental trial

**First author name:** Laurent Degroote

**Year of publication:** 2018

**Country of study:** Belgium

**Methodological quality assessment (Mixed Methods Appraisal Tool (MMAT), version 2018)**

| **Category of study designs** | **Methodological quality criteria** | **Response** | | | |
| --- | --- | --- | --- | --- | --- |
|  |  | **Yes** | **No** | **Can’t tell** | **Comments** |
| Screening questions (for all types) | S1. Are there clear research questions? | ✓ |  |  |  |
|  | S2. Do the collected data allow to address the research questions? | ✓ |  |  |  |
|  | *Further appraisal may not be feasible or appropriate when the answer is ‘No’ or ‘Can’t tell’ to one or both screening questions.* | | | | |
| 3. Quantitative nonrandomized | 3.1. Are the participants representative of the target population? | ✓ |  |  |  |
|  | 3.2. Are measurements appropriate regarding both the outcome and intervention (or exposure)? | ✓ |  |  |  |
|  | 3.3. Are there complete outcome data? |  | ✓ |  |  |
|  | 3.4. Are the confounders accounted for in the design and analysis? | ✓ |  |  |  |
|  | 3.5. During the study period, is the intervention administered (or exposure occurred) as intended? |  |  | ✓ |  |

**Article information**

**Article title:** Long-term effectiveness of a smartphone app for improving healthy lifestyles in general population in primary care: Randomized controlled trial (Evident II Study)

**First author name:** Luis Garcia-Ortiz

**Year of publication:** 2018

**Country of study:** Spain

**Methodological quality assessment (Mixed Methods Appraisal Tool (MMAT), version 2018)**

| **Category of study designs** | **Methodological quality criteria** | **Response** | | | |
| --- | --- | --- | --- | --- | --- |
|  |  | **Yes** | **No** | **Can’t tell** | **Comments** |
| Screening questions (for all types) | S1. Are there clear research questions? | ✓ |  |  |  |
|  | S2. Do the collected data allow to address the research questions? | ✓ |  |  |  |
|  | *Further appraisal may not be feasible or appropriate when the answer is ‘No’ or ‘Can’t tell’ to one or both screening questions.* | | | | |
| 2. Quantitative  randomized controlled  trials | 2.1. Is randomization appropriately performed? | ✓ |  |  |  |
|  | 2.2. Are the groups comparable at baseline? | ✓ |  |  |  |
|  | 2.3. Are there complete outcome data? | ✓ |  |  |  |
|  | 2.4. Are outcome assessors blinded to the intervention provided? |  |  | ✓ |  |
|  | 2.5 Did the participants adhere to the assigned intervention? |  | ✓ |  |  |

**Article information**

**Article title:** Implementation of the SMART MOVE intervention in primary care: a qualitative study using normalisation process theory

**First author name:** Liam G Glynn

**Year of publication:** 2018

**Country of study:** Ireland

**Methodological quality assessment (Mixed Methods Appraisal Tool (MMAT), version 2018)**

| **Category of study designs** | **Methodological quality criteria** | **Response** | | | |
| --- | --- | --- | --- | --- | --- |
|  |  | **Yes** | **No** | **Can’t tell** | **Comments** |
| Screening questions (for all types) | S1. Are there clear research questions? | ✓ |  |  |  |
|  | S2. Do the collected data allow to address the research questions? | ✓ |  |  |  |
|  | *Further appraisal may not be feasible or appropriate when the answer is ‘No’ or ‘Can’t tell’ to one or both screening questions.* | | | | |
| 1. Qualitative | 1.1. Is the qualitative approach appropriate to answer the research question? | ✓ |  |  |  |
|  | 1.2. Are the qualitative data collection methods adequate to address the research question? | ✓ |  |  |  |
|  | 1.3. Are the findings adequately derived from the data? | ✓ |  |  |  |
|  | 1.4. Is the interpretation of results sufficiently substantiated by data? | ✓ |  |  |  |
|  | 1.5. Is there coherence between qualitative data sources, collection, analysis and interpretation? | ✓ |  |  |  |

**Article information**

**Article title:** Process evaluation of an eHealth intervention implemented into general practice: General practitioners’ and patients’ views

**First author name:** Louise Poppe

**Year of publication:** 2018

**Country of study:** Belgium

**Methodological quality assessment (Mixed Methods Appraisal Tool (MMAT), version 2018)**

| **Category of study designs** | **Methodological quality criteria** | **Response** | | | |
| --- | --- | --- | --- | --- | --- |
|  |  | **Yes** | **No** | **Can’t tell** | **Comments** |
| Screening questions (for all types) | S1. Are there clear research questions? | ✓ |  |  |  |
|  | S2. Do the collected data allow to address the research questions? | ✓ |  |  |  |
|  | *Further appraisal may not be feasible or appropriate when the answer is ‘No’ or ‘Can’t tell’ to one or both screening questions.* | | | | |
| 1. Qualitative | 1.1. Is the qualitative approach appropriate to answer the research question? | ✓ |  |  |  |
|  | 1.2. Are the qualitative data collection methods adequate to address the research question? | ✓ |  |  |  |
|  | 1.3. Are the findings adequately derived from the data? | ✓ |  |  |  |
|  | 1.4. Is the interpretation of results sufficiently substantiated by data? | ✓ |  |  |  |
|  | 1.5. Is there coherence between qualitative data sources, collection, analysis and interpretation? | ✓ |  |  |  |
| 4. Quantitative  descriptive | 4.1. Is the sampling strategy relevant to address the research question? | ✓ |  |  |  |
|  | 4.2. Is the sample representative of the target population? | ✓ |  |  |  |
|  | 4.3. Are the measurements appropriate? | ✓ |  |  |  |
|  | 4.4. Is the risk of nonresponse bias low? |  |  | ✓ |  |
|  | 4.5. Is the statistical analysis appropriate to answer the research question? | ✓ |  |  |  |
| 5. Mixed methods | 5.1. Is there an adequate rationale for using a mixed methods design to address the research question? | ✓ |  |  |  |
|  | 5.2. Are the different components of the study effectively integrated to answer the research question? | ✓ |  |  |  |
|  | 5.3. Are the outputs of the integration of qualitative and quantitative components adequately interpreted? | ✓ |  |  |  |
|  | 5.4. Are divergences and inconsistencies between quantitative and qualitative results adequately addressed? | ✓ |  |  |  |
|  | 5.5. Do the different components of the study adhere to the quality criteria of each tradition of the methods involved? |  |  | ✓ |  |

**Article information**

**Article title:** Development and efficacy of an electronic, culturally adapted lifestyle counseling tool for improving diabetes- related dietary knowledge: Randomized controlled trial among ethnic minority adults with type 2 diabetes mellitus

**First author name:** Kathleen Abu-Saad

**Year of publication:** 2019

**Country of study:** Israel

**Methodological quality assessment (Mixed Methods Appraisal Tool (MMAT), version 2018)**

| **Category of study designs** | **Methodological quality criteria** | **Response** | | | |
| --- | --- | --- | --- | --- | --- |
|  |  | **Yes** | **No** | **Can’t tell** | **Comments** |
| Screening questions (for all types) | S1. Are there clear research questions? | ✓ |  |  |  |
|  | S2. Do the collected data allow to address the research questions? | ✓ |  |  |  |
|  | *Further appraisal may not be feasible or appropriate when the answer is ‘No’ or ‘Can’t tell’ to one or both screening questions.* | | | | |
| 2. Quantitative  randomized controlled  trials | 2.1. Is randomization appropriately performed? | ✓ |  |  |  |
|  | 2.2. Are the groups comparable at baseline? | ✓ |  |  |  |
|  | 2.3. Are there complete outcome data? | ✓ |  |  |  |
|  | 2.4. Are outcome assessors blinded to the intervention provided? |  | ✓ |  |  |
|  | 2.5 Did the participants adhere to the assigned intervention? | ✓ |  |  |  |

**Article information**

**Article title:** The HealtheSteps^TM^ lifestyle prescription program to improve physical activity and modifiable risk factors for chronic disease: a pragmatic randomized controlled trial

**First author name:** D. P. Gill

**Year of publication:** 2019

**Country of study:** Canada

**Methodological quality assessment (Mixed Methods Appraisal Tool (MMAT), version 2018)**

| **Category of study designs** | **Methodological quality criteria** | **Response** | | | |
| --- | --- | --- | --- | --- | --- |
|  |  | **Yes** | **No** | **Can’t tell** | **Comments** |
| Screening questions (for all types) | S1. Are there clear research questions? | ✓ |  |  |  |
|  | S2. Do the collected data allow to address the research questions? | ✓ |  |  |  |
|  | *Further appraisal may not be feasible or appropriate when the answer is ‘No’ or ‘Can’t tell’ to one or both screening questions.* | | | | |
| 2. Quantitative  randomized controlled  trials | 2.1. Is randomization appropriately performed? | ✓ |  |  | Trial retention was 76% at 6 months |
|  | 2.2. Are the groups comparable at baseline? | ✓ |  |  |  |
|  | 2.3. Are there complete outcome data? |  | ✓ |  |  |
|  | 2.4. Are outcome assessors blinded to the intervention provided? |  | ✓ |  |  |
|  | 2.5 Did the participants adhere to the assigned intervention? | ✓ |  |  |  |
