# Supplementary material for: Usability and utility of eHealth for physical activity counselling in primary health care: a scoping review
Source: BMC Fam Pract. 2020 Nov 6;21:229. doi: 10.1186/s12875-020-01304-9 (PMC7648312; doi:10.1186/s12875-020-01304-9)
Supplement: Supplementary file 2 — Additional file 2. Summary of the included studies [file 12875_2020_1304_MOESM2_ESM.docx]

**Additional File 2** Summary of the included studies

| Authors, year, country | MMAT score | Study design | Participant and setting | Counselling domain | eHealth used and process | Outcomes |
| --- | --- | --- | --- | --- | --- | --- |
| Prochaska et al.,  2000, USA | 4/5 | Cross-sectional study | **Participant:**  - 252 adolescents (aged 11-18 years)  - 285 adults  - 28 providers and office staff  **Setting:**  - 12 primary care clinics | - PA  - Nutrition  (Patient-centred Assessment and Counseling for Exercise plus Nutrition (PACE+)) | - Personal computer  - Internet (web-based programme: HyperText Markup Language (HTML) and JavaScript, run on Netscape Navigator)  - 30-45 minutes during waiting time  - Colour printouts for both patients and providers | **Usability:**  - > 90% liked the look and feel of the programme  - > 90% understood the words and ideas  - > 85% felt comfortable to enter personal information  **Utility:**  - About 70% believed the information fit well or very well  - Nearly all adults (92%) evaluated the appropriate length of the programme  - 21% of adolescents rated the programed as too long  - Adults: action plan counselling = 5.4 min  - Adolescents: action plan counselling = 8.6 min  - 68% of adolescents and 45% of adults rated the programme and printouts as helpful or very helpful  - 64% of adolescents and 45% of adults rated the provider interaction as high  - 85% of staff perceived the programme as helpful or very helpful  - 72% of staff reported being satisfied or very satisfied  - 77% of staff would recommend the programme to other primary care offices |
| Calfas et al., 2002, USA | 4/5 | Four-group intervention study | **Participant:**  - 173 adult patients  - 10 physicians and nurse practitioners  **Setting:**  - 4 primary care settings | - PA  - Nutrition  - Dietary fat  - Fruits/  vegetables  - Overeating behaviours  (PACE+) | - Computer programme assessed the target behaviours and guided participants to develop the tailor action plans  - Printed the participants’ behavioural change goals for both patients and providers  - 2-5 min review and counselling by providers  - 4 follow-up methods (control, mail, infrequent phone and mail, frequent phone and mail) | **Usability:**  - Providers satisfied the programme and protocol (4.1/5)  **Utility:**  - Almost half of patients rated the computer programme and provider counselling as helpful or very helpful  - Providers rated the printout as helpful in discussing PA (4.1/5)  - Providers reported the programme and counselling help their patients (3.8/5)  - Providers would continue using the programme (3.9/5)  - Providers would recommend the programme to other primary care providers (3.8/5) |
| Pinto et al., 2002, USA | 3/5 | Randomised controlled trial | **Participant:**  - 298 sedentary adults (intervention group: 150 and comparison: 148)  **Setting:**  a large multisite, multispecialty group practice | Intervention: PA  Comparison: nutrition | - Telephone linked communication used computer technology and digitised human speech (automated telephone conversations)  - Participants used telephone keypads to communicate  - About 10 min/call  - Participants were asked to call every week for the first 3 months, and at least biweekly until 6 months | **Usability:**  - Some participants preferred a human counsellor (technical problems made difficulty) and needed reminders to call  - Usability score = 3.82/5  **Utility:**  - Significant effects on energy expenditure in moderate-intensity PA at 3 months (p = 0.02)  - More likely to meet the recommended level of MVPA at 3 months (p = 0.02)  - More advanced stages of motivational readiness (action and maintenance) (p = 0.04)  - Effects on reminding and tracking healthy behaviours score = 3.4/5  - Effects on the motivational impact score = 2.91/5 |
| Anhøj et al., 2004, Denmark | 3/5 | Qualitative study (semi-structured interviews) | **Participant:**  - 5 GPs  - 25 adult patients  **Setting:**  - General practice | - PA  - Diet (main) | - Internet based programme (required username and password)  - 219 diet questions took about 30 min  - PA questions covered 9 different activity levels from sleep to hard PA (took a few minutes to complete)  - Results that summarised energy intake, energy expenditure, and composition of diet were generated  - The patient selected 1 of 5 offered diet and 1 of 3 PA programmes based on motivations  - The programme generated email with the results of the questionnaire and personalised advice  - After 4 weeks, patients received an E-mail asking them to update answers and received the new advice based on the current lifestyle change program | **Usability:**  - GPs cited the programme was complicated and too long.  - Patients noted the programme was complicated  **Utility:**  - GPs stated some errors of the programme, and the programme was unable to sustain the patient's attention due to the complexity of the programme  - Patients required more interactions between the programme and patients |
| Sciamanna et al., 2004, USA | 11/15 | Mixed methods study | **Participant:**  - 10 primary care physicians  **Setting:**  - 10 public health clinics | - PA  - Smoking | - Computer-tailored health communication programmes  - Patients entered their data (using graphical user interface)  - The programme collected data and provided feedback based on the PA variables  - Smoking variables were collected and feedback was provided  - 10 min to complete | **Usability:**  - Inexperienced staff to use the programme  - Some technical problems with the computer and/or printer  **Utility:**  - 9 out of 10 clinics did not utilise the programme  - Inconsistent with practice workflow  - An additional time burden |
| Carlfjord et al., 2009, Sweden | 4/5 | Cross-sectional study | **Participant:**  - 3027 patients  **Setting:**  - 9 PHC units | - PA  - Alcohol consumption | - Computer-based lifestyle intervention  - Alcohol consumption questions collected beverage-specific, weekly consumption and frequency of heavy episodic drinking  - PA questions measured number of days/week with moderate-intensity and vigorous-intensity aerobic PA for a minimum of 30 min and motivation towards PA  - A printed sheet provided information on risk levels and tailored advice based on input answers | **Usability:**  - 88% stated easy or very easy to complete the programme  **Utility:**  - 84% felt positive with the referral to the programme |
| Carlfjord et al., 2010, Sweden | 4/5 | Cross-sectional study | **Participant:**  - 291 PHC staff (GPs, nurses, and others) (172 responders, 59%)  **Setting:**  - 9 PHC units | - PA  - Alcohol consumption | - Computer-based lifestyle intervention  - Staff addressed lifestyle issues of patients and referred patients to perform the computer-based test within the PHC  - Patients received written tailored advice generated by the computer  - Patients decided whether to discuss the results with a staff | **Usability:**  - 50% agreed or partially agreed ‘I feel I have been involved in the process of introducing the computer-based test at this’  - 73% agreed or partially agreed ‘To use the computer-based test is well accepted in the staff group’  - 59% agreed or partially agreed ‘We discuss the computer-based test in the staff group’  - 66% agreed or partially agreed ‘Due to the computer-based test more patients also receive verbal advice about alcohol or physical activity’  - 61% agreed or partially agreed ‘It has become easier to address lifestyle issues in patient contacts since the computer-based test was introduced’  **Utility:**  - 85% agreed or partially agreed ‘I find it positive to refer patients to perform the test’  - 93% agreed or partially agreed ‘I agree with the advice provided by the computer’  - 75% agreed or partially agreed ‘My opinion is that the computer-based concept could have an effect on patients’ lifestyle’  - 78% agreed or partially agreed ‘I have confidence in the computer-based test’ |
| Carroll et al., 2010, USA | 3/5 | Randomised controlled trial | **Participant:**  - 394 patients (intervention group: 187 and control group: 207) (41 dropouts: 22 intervention; 19 control)  **Setting:**  - A family medicine practice | Intervention: exercise  Comparison: prevention (behavioural risk factor surveillance and feedback reports) | - Computerised tailored report  - Patients were asked about current PA habits, self-effıcacy, decision making about PA, health status, and chronic conditions  - Staff entered the participant’s responses into the computer programme  - A personalised report was generated and mailed to the patient, designed to motivate patients to increase PA  - Patients did the surveys at baseline, 1, 3, and 6 months | **Usability:**  - N/A  **Utility:**  - The increase in PA between intervention and control groups were not significant (139 min/week VS 103 min/week, p = 0.45) [main outcome] – PA increased from baseline, but the change was not significant compared to the control group  - Improvements in overcoming barriers and consideration of  benefıts to exercise (behavioural and cognitive processes) |
| Becker et al., 2011, Germany | 10/15 | Mixed methods study (pre- and post-intervention study and qualitative interviews) | **Participant:**  - 79 patients with either coronary heart disease, diabetes, or both  **Setting:**  - 5 general practices | - PA | - Computer-based counselling system  - The content was based on the transtheoretical model (TTM); and self-efficacy from social cognitive theory and affective associations toward PA  - The counselling system was installed on Tablet PCs with built-in speakers and touch sensitive  - Patients answered questions  - The system classified the patient according the stage of change and provided description of the stage specific session content  - Each session took about 10 min | **Usability:**  - All patients could use the system without further  instructions from the researcher (perceived ease of use)  - Several patients required more time and privacy to use the system (perceived costs)  **Utility:**  - Positive change in affective and cognitive attitude components  - Change in self-efficacy was not significant  **-** Perceived usefulness of the system was debatable (different opinions emerged from the qualitative interviews) |
| Christian et al., 2011, USA | 5/5 | Prospective controlled  Trial (non-randomised) | **Participant:**  - 279 patients with metabolic syndrome (intervention group: 140 and control group: 139) (16 dropouts: 7 intervention; 9 control)  **Setting:**  - 2 large urban community-based health centres (with family practice physicians) | - PA  - Diet | - Computer support programme  - The programme assessed motivational readiness to increase PA and make dietary changes before a visit  - The programme took less than 10 min (90% of patients completed within 2 min with assistance from the study coordinator  - The system created a 4-5 pages individualised, tailored report  - The feedback and content were based on 4 theories: Transtheoretical Model of Change, motivational interviewing, social-cognitive theory, and decision making  - The system also generated a report for physicians, which consisted of a brief, bulleted summary of the findings, and provided the physician with patient-specific counseling recommendations | **Usability:**  - N/A  **Utility:**  - Changes in body weight were significantly different between the intervention (decreased 1.50 kg) and control (increased 0.15 kg) groups (p = 0.002) [primary outcome]  - A significant reduction in waist circumference among intervention group (decreased 2.2 cm) vs control group (increased 1.5 cm) (p = 0.01)  - A significant reduction in energy intake (14.9% vs 6.6%) (p = 0.02)  - No significant change in PA levels  - No significant change in secondary end points (i.e. fasting insulin, fasting glucose, total cholesterol, low-density lipoprotein cholesterol, high-density lipoprotein cholesterol, triglyceride levels, systolic and diastolic blood pressure) |
| Leijon et al., 2011, Sweden | 4/5 | Non-randomised trial | **Participant:**  - 311 physically inactive patients (patient-initiated group: 129 vs staff-referred group: 182)  **Setting:**  - 10 PHC units | - PA  - Alcohol consumption | - Electronic screening and brief intervention (a stand-alone information, touch-screen information technology kiosks)  - A personalised written feedback was generated, including the current PA level, and printed  out at the kiosk | **Usability:**  - N/A  **Utility:**  - Both groups had statistically significant PA scores (not significant between groups) |
| De Coker et al., 2012, Belgium | 3/5 | Randomised controlled trial | **Participant:**  - 92 patients (tailored condition: 45 (pedometer and computer-tailored step advice) and standard condition: 47 (pedometer only))  **Setting:**  - Recruitment from 38 general practices | - PA (step counts – including ambulatory activities, biking, and swimming (every minute of biking or swimming = 150 steps) | - Computer-tailored website (no patient-provider interaction)  - The programme assessed demographic information, baseline step level (collecting by pedometers 7 days prior to the assessment), and the psychosocial correlates of achieving 10,000 steps/day  - The tailored feedback was generated based on the inputs using the concepts of the theory of planned behavior and the transtheoretical model | **Usability:**  - 70% requested the computer-tailored step advice  - 100% rated the system as understandable  - 94% rated as credible  - 83% rated as relevant  - 72% rated not too long and instructive  - 67% rated as and encouraging to increase steps  **Utility:**  - Daily step counts increased significantly in both conditions, however, the standard condition was more effective (tailored condition: +1505 steps/day and standard condition: +4141 steps/day) |
| Parekh et al., 2012, Australia | 4/5 | Randomised controlled trial | **Participant:**  - 2306 patients (intervention group: 1199 and control group: 1107) (1711 (76%) completed the survey – intervention: 868; control: 843)  **Setting:**  - Recruitment from 21 general practices | - PA  - Smoking  - Alcohol  - Diet (i.e. meat, fish, fruits and vegetables, unsaturated fats as spreads, added salt, type of milk consumption, BMI) | - Personalised computer-tailored feedback (no patient-provider interaction)  - 26 baseline questions were asked to assess the Prudence Score (health behaviours score: 0-10)  - One-page health promotion material was distributed to participants only for unattainable behaviours  - 3-month follow-up to compare pre- and post- health behaviours score  - Control group: received an individualised letter and tailored information about other 5 health protective behaviours (sun protection, tetanus vaccination, mammogram and Pap smear) | **Usability:**  - N/A  **Utility:**  - Changes in PA scores were not statistically significant (intervention: +0.48 vs control: -1.34) (p = 1.06)  - Overall changes in health behaviours score were statistically significant (but no significant change in PA) |
| Casey et al., 2014, Ireland | 5/5 | Qualitative study | **Participant:**  - 12 participants  **Setting:**  - Primary care centres | - PA | - Smartphone app (Accupedo-Pro Pedometer app) provided automatic feedback and tracking of step  count and calories burnt as well as goal setting and feedback  - At 8 weeks, changes in step counts (primary outcome) and secondary outcomes (i.e. blood pressure, resting heart rate, body weight, BMI, emotions, quality of life) were compared between the intervention and control groups  - The qualitative interviews were conducted among some participants after the intervention (the SMART MOVE randomised controlled trial) | **Usability:**  - The app was easy to use  - Participants stated some challenges included an increase in battery consumption and the inconvenience to carry their phones to record step counts  **Utility:**  - The app facilitated positive changes through awareness and knowledge, goal setting and feedback |
| Glynn et al., 2014, Ireland | 4/5 | Randomised controlled trial | **Participant:**  - 90 participants  (intervention group: 45 and control group: 45) (intervention: app use and control: encouragement to walk 30 min/day)  **Setting:**  - 3 primary care centres | - PA | - Smartphone app (Accupedo-Pro Pedometer app) provided automatic feedback and tracking of step  count and calories burnt as well as goal setting and feedback  - At 8 weeks, changes in step counts (primary outcome) and secondary outcomes (i.e. blood pressure, resting heart rate, body weight, BMI, emotions, quality of life) were compared between the intervention and control groups | **Usability:**  - N/A  **Utility:**  - The mean differences of step counts (week 1 and week 8) were statistically significant between the intervention group (+1631 steps/day) and the control group (-386 steps/day) (p=0.025) [primary outcomes]  - All the secondary outcomes were not significantly difference between the groups |
| Parekh et al., 2014, Australia | 4/5 | Randomised controlled trial  (4 groups: intervention – (i) single contact and (ii) dual contact; control: (iii) single contact; and (iv) dual contact) | **Participant:**  - 3065 patients (2873 completed the survey)  **Setting:**  - Recruitment from 21 general practices | - PA  - Smoking  - Alcohol  - Diet (i.e. meat, fish, fruits and vegetables, unsaturated fats as spreads, added salt, type of milk consumption, BMI) | - Personalised computer-tailored feedback (no patient-provider interaction)  - 26 baseline questions were asked to assess the Prudence Score (health behaviours score: 0-10)  - One-page health promotion material was distributed to participants only for unattainable behaviours  - Control group: received an individualised letter and tailored information about other 5 health protective behaviours (sun protection, tetanus vaccination, mammogram and Pap smear)  - 12-month follow-up to compare pre- and post- overall health behaviours score, and percentage changes in adherence to each health behaviour | **Usability:**  - N/A  **Utility:**  - The percentage changes in adherence to PA from baseline to at 12 months were not significant between intervention and control groups (single contact: -0.5% vs –3.1%, p = 0.12; dual contact: -1.2 vs -6.2, p = 0.10)  - The significant changes were found for alcohol and some dietary behaviours |
| Verwey et al., 2014, Netherlands | 3/5 | Qualitative study  (interviews: patients and a focus group discussion: nurses) | **Participant:**  - 20 patients (aged > 40 years) with chronic illnesses  **ce**  **Setting:**  - 2 primary care practices | - PA  (It's LiFe!) | - Iterative user-centred mobile technology (with face-to-face consultations)  - The tool connects 3 technologies: an accelerometer, a smartphone app, and an internet app  - The tool focused on self-monitoring, goal-  setting, and feedback  - The counselling protocol was based on the ‘Five A’s’ model,  Theory of Planned Behavior, the Goal-Setting Theory, the Self-Determination Theory, and Motivational Interviewing  - 3 face-to-face counselling sessions were conducted by nurses  - Between the 2^nd^ and 3^rd^ consultations, the tool had been used for 2-3 months to monitor PA and gave automated feedback | **Usability:**  - Participants (12 out of 17) were positive about the tool (felt encouraged to be active)  - Positive feedback was given on consultations  - 18 out of 20 patients mentioned technical problems  - All nurses agreed on the usefulness of objective PA measurement  - The nurses received a lot of queries due to technical problems (‘they spent more time explaining the tool than on physical activity counseling’)  **Utility:**  - 12 patients were positive about the effect of the intervention on their PA and 5 patients were neutral  - > 50% stated the added value of the consultations and the involvement of nurses  - The calculation of 13 patients, mean PA time significantly increased by 10.6 min/day (p = 0.02)  - The nurses mentioned it was much easier to talk about barriers and facilitators when saw the data together |
| van der Weegen et al., 2015, Netherlands | 5/5 | Three-arm clustered randomized controlled trial - (i) tool and SSP; (ii) SSP; and (iii) care as usual | **Participant:**  - 199 inactive patients (aged 40-70 years) with T2DM or COPD (group 1: 65; group 2: 66; and group 3: 68)  **Setting:**  - 24 general practices | - PA  (It's LiFe!) | - The tool consisted of a three-dimensional (3D) activity monitor,  a mobile phone app, and a web app  - Automated feedback messages for personal goal were sent  - Interactions (answers and dialogues) between participants and the tool were seen by the nurses via the web app  - Self-management support programme (SSP) was 4 individual consultations conducted by nurses in the first week, after 2 weeks, after 2-3 months, and after  4-6 months | **Usability:**  - N/A  **Utility:**  - Participants who received the tool and the SSP had 8 min more MVPA than participants in the SSP, and 12 min more PA than the care as usual group  - At 3 months after the end of the intervention, this improvement difference was 9 min (SSP) and 11 min (care as usual)  - For secondary outcomes, general-efficacy and exercise-efficacy were not different significantly among three groups  - For quality of life (another secondary outcome), physical component scores were significantly higher in the SSP group compared with tool and SSP, and care as usual; mental component scores were significantly higher in both intervention groups (tool and SSP, and SSP) compared with the care as usual group |
| Choo et al., 2016, Korea | 4/5 | Pre-post single-group design | **Participant:**  - 30 patients with obesity  **Setting:**  - Family medicine clinic | - Weight reduction (main)  - PA | - Mobile app linked with an accelerometer consisted of 4 main features: (i) personalised educational materials; (ii) personal goal setting for weight loss, (iii) self-monitoring of weight and PA linked with an accelerometer, and (iv) online social support from peers and web-based communication with clinicians  - Body weight and abdominal circumference were measured before and after the study period (1 month) | **Usability:**  - Most usability aspects (6 out of 9 aspects) were reported high rates by 50% of participants (≥ 66.66%: 3 out of 9 aspects)  - All acceptability aspects (15 out of 15) were reported high rates by 50% of participants (≥ 66.66%: 9 out of 15 aspects)  - Scales of the depth of patient-doctor relationship significantly decreased (p = 0.02)  **Utility:**  - Most participants achieved more than their recommended exercise (median 125.9% of target exercise goal)  - Mean weight change was not significant period  - Mean abdominal circumference was significantly reduced (-1.84 cm) |
| Diaz et al., 2016, USA | 4/5 | Quasi-experimental study | **Participant:**  - 252 adults (aged 18-35 years) (intervention group: 105; control group: 96)  **Setting:**  - 2 PHC practices (federally qualified health centres) | - PA  - Nutrition  - Weight  - Smoking  - Alcohol | - Tablet-based risk assessment programme  - The programme provided an interactive questionnaire and feedback  - The programme provided  immediate feedback regarding a particular unhealthy behaviour and to talk with healthcare providers  - The programme generated a  positive statement for their healthy  behaviours with information about their importance | **Usability:**  - 2 out of 3 aspects regarding participant satisfaction with providers during the first week follow-up were significantly higher in the intervention group  **Utility:**  - No significant differences of accurate views (perceived vs actual) of behaviours between the intervention and control groups.  - Among obese and overweight participants, the intervention group was more likely to discuss weight loss with doctors (p = 0.0088) (1 out of 5 aspects was significant; for PA domain, the results were not significant) |
| Mann et al., 2016, USA | 4/5 | Randomised controlled trial | **Participant:**  - 54 patients with prediabetes (intervention group: 27 and control group: 27)  **Setting:**  - 2 academic primary care practices | - PA  - Diet  (Avoiding Diabetes Thru Action Plan Targeting (ADAPT)) | - Shared goal-setting tool embedded in EMR  - The tool alerted providers about  Previous diet and PA which the patient was willing to change  - The EMR-embedded action planning tool helped guide a conversation about lifestyle change  - Pedometers were used to measure PA  - The results were assessed at 6 months | **Usability:**  - N/A  **Utility:**  - Total daily steps were higher in the intervention group compared with the control group (+1418 vs −598, p = 0.007)  - The 7-day average steps were higher in the intervention group compared with the control group (+1345 vs −646, p = 0.01) |
| Recio-Rodriguez et al., 2016, Spain | 3/5 | Randomised controlled trial | **Participant:**  - 833 participants (intervention group (counselling and app): 415 and control group (counselling): 418)  **Setting:**  - 6 primary care centres in family practice offices | - PA  - Diet (Mediterranean) | - Mobile phone app designed to promote the Mediterranean diet and increase PA  - The participants entered their food intake and recorded their PA using the accelerometer of the device, and user input of activities performed without the mobile phone (e.g. swimming)  - The app reported a daily summary of food intake and PA performed and a balance of energy  - The app generated a  recommended plan for the following days  - Follow-up at 3 months | **Usability:**  - N/A  **Utility:**  - No significant differences in increase of PA were found  between the two groups  - Participants who adhered to the app (used the app > 60 days) showed an increase in moderate PA (42.9 min/week, 95%CI 1.8-83.9) and MVPA (44.0 min/week, 95% CI 2.1-86.0) and a decrease in sedentary time (126.1 min/week, 95% CI 18.9-233.4) |
| Verwey et al., 2016, Netherlands | 10/15 | Mixed methods study  (interviews and randomised control trial) | **Participant:**  - 20 practice nurses  - 131 physically inactive patients with COPD or T2DM (intervention group: tool and consultations; control group: consultations)  **Setting:**  - 16 family practices | - PA  (It's LiFe!) | - The tool consists of an accelerometer, a smartphone app, and a web app  - Patients received personalised feedback  - Both groups received 4 consultations | **Usability:**  - 88% of the patients used the tool until the end of the intervention  - Most patients were positive about the tool  - 58% of the patients experienced problems using the tool  - 90% of the nurses inspected results from the web app during consultations  - 50% of the nurses inspected results from the web app between each consultation  - The nurses indicated the tool encouraged people to be more physically active  - The nurses monitored their patients through the web app  **Utility:**  - N/A |
| Walters et al., 2017, UK | 3/5 | Cross-sectional study | **Participant:**  - 526 participants aged ≥ 65 years (34% of the population) (454 (86.3%) completed the questionnaires)  **Setting:**  - 5 general practices | - Health and social risks (including PA, nutrition, smoking, alcohol) | - Tailored computer-aided health and social risk appraisal system  - Participants answered the postal questionnaire, including, health, lifestyle, social and environmental domains  - The data from the questionnaires were entered  - The software system generated a personalised feedback report with advice on health and wellbeing  - Participants with new concerning or complex needs were advised to follow-up at the general practices | **Usability:**  - N/A  **Utility:**  - The system was feasible for general practices to implement, yield useful information, and identify individual needs (24% were followed up by providers)  - Participation rates were low (34% of the population)  - The intervention cost was £125 (196 USD) per practice in the first year, and then £70 (110 USD) per practice in subsequent years (£6.71 (10.50 USD) per patient for every 1000 patients receiving the questionnaire) |
| Degroote et al., 2018, Belgium | 3/5 | Quasi-experimental study | **Participant:**  - 310 allocated participants (intervention group: 161; control group: 149) (32.2% completed the entire intervention)  **Setting:**  - 19 general practices | - PA  - Nutrition (fruit and vegetable consumption)  (MyPlan 1.0) | - Website to promote healthy behaviour  - The programme guided different self-regulation techniques (tailored feedback, action planning, coping planning, and self-monitoring  - The follow-up period was at 1 week and 1 month (control group: received general feedback) | **Usability:**  - N/A  **Utility:**  - Total PA and MVPA were increased in the intervention group (decreases in total PA and MVPA were found in the control group)  - There was no intervention effect on vigorous-intensity PA |
| Garcia-Ortiz et al., 2018, Spain | 3/5 | Randomised controlled trial | **Participant:**  - 833 participants (intervention group (counselling and app): 415 and control group (counselling): 418) (intervention: 352 vs control: 363 completed the 12-month follow-up)  **Setting:**  - 6 primary care centres | - PA  - Diet (Mediterranean) | - Smartphone app designed to promote the Mediterranean diet and increase PA  - The participants entered their food intake and recorded their PA using the accelerometer of the device, and user input of activities performed without the mobile phone (e.g. swimming)  - The app reported a daily summary of food intake and PA performed and a balance of energy  - The app generated a  recommended plan for the following days  - Follow-up at 12 months | **Usability:**  - N/A  **Utility:**  - There were no positive intervention effects on PA and diet (comparisons between the intervention and control groups)  - Within the intervention group, high app adherence participants (used the app > 60 days) had significantly different MVPA (compared with the low adherence), however, the MVPA in both high and low app adherence were decreased from the baseline |
| Glynn et al., 2018, Ireland | 5/5 | Qualitative study (focus group discussion – providers and interviews – service users) | **Participant:**  - 14 primary care providers  - 4 service users  **Setting:**  - 4 primary care centres | - PA | - Smartphone app (Accupedo-Pro Pedometer app) provided automatic feedback and tracking of step  count and calories burnt as well as goal setting and feedback  - At 8 weeks, changes in step counts (primary outcome) and secondary outcomes (i.e. blood pressure, resting heart rate, body weight, BMI, emotions, quality of life) were compared between the intervention and control groups  - The qualitative interviews were conducted among some participants after the intervention (the SMART MOVE randomised controlled trial)  - This qualitative study evaluated the potential barriers and levers to the implementation of the intervention | **Usability:**  - The app was easy to use  - Some participants (both providers and service users) highlighted technological issues as the challenges  **Utility:**  - The app supported both providers and users to promote PA  - The tool promoted motivations towards PA among providers and service users  - Some providers concerned about extra workload |
| Poppe et al., 2018, Belgium | 13/15 | Mixed methods study | **Participant:**  - 232 patients completed the survey  - 15 GPs participated in the interviews  **Setting:**  - 19 general practices | - PA  - Nutrition (fruit and vegetable consumption)  (MyPlan 1.0) | - Online programme based on self-regulation theory  - Users completed the questionnaire  - The programme created personal advice and a specific action plan  - Follow-up at 1 week and 1 month | **Usability:**  - There were inconsistent opinions among GPs. For example, some GPs did not believe that using eHealth was a good approach, while some GPs supported using eHealth  **Utility:**  - 72% of patients indicated general practice as a feasible setting to implement the intervention  - 73.3% of patients did not discuss the intervention with GPs  - All patients did not discuss their personal advice or action plan with GPs  - GPs raised some barriers and facilitators to implement the intervention (it meant there were pros and cons) |
| Abu-Saad et al., 2019, Israel | 4/5 | Randomised controlled trial | **Participant:**  - 50 patients with T2DM (aged 40-64 years) (intervention group: 25 and control group: 25)  **Setting:**  - Primary care clinics | - Diet (main)  - PA  (Interactive Lifestyle Assessment, Counseling, and Education (I-ACE)) | - Computer software was designed for use by dieticians to support clinical lifestyle counseling  - The software collected data on habitual dietary and PA behaviours and calculated summary measures – - The software supported the dietician-patient team in building and tracking a tailored healthy lifestyle programme  - The control group received the standard lifestyle advice  - Both groups received 4 individual dietary counseling sessions at clinics | **Usability:**  - Most participants thought that the software was helpful to the dietary counseling (91%) and did not detract from their interaction with the dietician (96%)  **Utility:**  - No significant difference of leisure PA between the intervention and control groups (p = 0.16) |
| Gill et al., 2019, Canada | 3/5 | Randomised controlled trial | **Participant:**  **-** 118 participants at risk or diagnosed with a chronic disease (intervention group: 59 and control group: 59)  **Setting:**  - 5 primary care/health care services sites | - PA  - Diet | - Customised health technology (eHealth) tools consisted of (i) phone coaching, (ii) online social network, (iii) smartphone app with a virtual coach and step counter, and (iv) website  - The intervention group participated in 3 phases: (i) 0-6 months – in-person coaching and eHealth use; (ii) 7-12 months - eHealth use; and (iii) 13-18 months – eHealth without phone coaching and social network  - The control group was encouraged to continue usual activities | **Usability:**  - N/A  **Utility:**  - The mean step counts at 6 months in the intervention group was 3132 steps/day more than the control group (p < 0.001) [primary outcome]  - The mean sitting time at 6 months of the intervention group was 0.08 min/day lower than the control group (p = 0.03) [secondary outcome]  - Other secondary outcomes related to PA were not statistically significant |

*App* application, *BMI* body mass index, *COPD* chronic obstructive pulmonary disease, *EMR* electronic medical record, *GP* general practitioner, *MMAT* Mixed Methods Appraisal Tool, *MVPA* moderate- and vigorous-intensity physical activity, *N/A* not available, *PHC* primary health care, *PA* physical activity, *T2DM* type 2 diabetes mellitus
